# Contributions of the maternal oral and gut microbiome to placental microbial colonization in overweight and obese pregnant women

**DOI:** 10.1038/s41598-017-03066-4

**Published:** 2017-06-06

**Authors:** Luisa F. Gomez-Arango, Helen. L. Barrett, H. David McIntyre, Leonie K. Callaway, Mark Morrison, Marloes Dekker Nitert

**Affiliations:** 1 Faculty of Medicine, The University of Queensland, Brisbane, Australia; 2 UQ Centre for Clinical Research, The University of Queensland, Brisbane, Australia; 3 Obstetric Medicine, Royal Brisbane and Women’s Hospital, Brisbane, Australia; 4 Mater Research, The University of Queensland, Brisbane, Australia; 5 Diamantina Institute, Faculty of Medicine and Biomedical Sciences, The University of Queensland, Brisbane, Australia; 6 School of Chemistry and Molecular Biosciences, The University of Queensland, Brisbane, Australia

## Abstract

A distinct bacterial signature of the placenta was reported, providing evidence that the fetus does not develop in a sterile environment. The oral microbiome was suggested as a possible source of the bacterial DNA present in the placenta based on similarities to the oral non-pregnant microbiome. Here, the possible origin of the placental microbiome was assessed, examining the gut, oral and placental microbiomes from the same pregnant women. Microbiome profiles from 37 overweight and obese pregnant women were examined by 16SrRNA sequencing. Fecal and oral contributions to the establishment of the placental microbiome were evaluated. Core phylotypes between body sites and metagenome predictive functionality were determined. The placental microbiome showed a higher resemblance and phylogenetic proximity with the pregnant oral microbiome. However, similarity decreased at lower taxonomic levels and microbiomes clustered based on tissue origin. Core genera: *Prevotella, Streptococcus* and *Veillonella* were shared between all body compartments. Pathways encoding tryptophan, fatty-acid metabolism and benzoate degradation were highly enriched specifically in the placenta. Findings demonstrate that the placental microbiome exhibits a higher resemblance with the pregnant oral microbiome. Both oral and gut microbiomes contribute to the microbial seeding of the placenta, suggesting that placental colonization may have multiple niche sources.

## Introduction

The establishment of the early microbiome in neonates influences infant growth and immune function. Recently, the maternal microbiome has been shown to prepare the newborn for host-microbial symbiosis, driving postnatal innate immune development^1^. Perturbations of infant microbial colonization have been associated with increased risk of asthma and obesity^2–4^. It has recently been suggested that intestinal microbial colonization may be initiated *in utero*, possibly by distinct bacteria present in the placenta and amniotic fluid^5^. Mounting evidence supports the presence of bacterial DNA in the placenta, raising questions on the potential role of intrauterine bacteria in placental function and fetal development.

Diverse hypotheses have been proposed for the mechanism of placental colonization: Vertical translocation from the vagina^6^, or hematogenous spread from the gut^7^ and the oral cavity^8^. Hematogenous spread from the oral cavity has received a wider attention due to the known association of periodontal disease with preterm birth^9^. The largest metagenomic study characterizing placental microbial communities demonstrated that the placental microbiome appears to be different from those of other body sites, albeit with some similarities to the oral non-pregnant microbiome^10^. However, the composition of the placental microbiome has not yet been prospectively compared within the same pregnant woman, during the same pregnancy, and with samples of the microbiomes at other body sites. This research is essential, to assess how the placental microbiome might be initiated and develop.

The purpose of this study was to assess similarities of the placental microbiome with other maternal microbiomes within the same individual. To our knowledge, this is the first study to address the origin of the placental microbiome by examining oral, gut and placental samples from the same pregnant women. Microbiome profiles from these three different tissue compartments were collected from women enrolled in the SPRING cohort^11^.

## Results

### Study population

Maternal characteristics from the 37 overweight (n = 13) and obese mothers (n = 24) included in this substudy are presented in Table 1. By design, women who delivered preterm, developed gestational diabetes mellitus or preeclampsia were excluded from this substudy. All women were of Caucasian ethnicity and the majority delivered vaginally.

**Table 1:** Maternal clinical characteristics.

*Mother* (*n* = *37*)	
Age (years)	34.5 (30.3–36.8)
BMI (kg/m^2^)	31.5 (27.9–35.8)
Blood pressure (mmHg)	
*Systolic*	108.0 (105.0–115.0)
*Diastolic*	69.0 (62.0–73.5)
Gestational age at delivery (wks)	39.4 (38.6–40.3)
Mode of delivery	
*Vaginal*	54.1%
*Cesarean*	45.9%
Antibiotic at delivery	
*Yes*	48.6%
*No*	51.4%
Gestational weight gain (kg)	8.3 (5.8–12.3)
Birth weight (g)*	3589 (3210–3946)
Gender*	
*Male*	67.7%
*Female*	32.3%

Clinical characteristics of mother-baby dyads. All data is presented as median with 25–75^th^ interquartile range. *Data available from only 31 infants.

### Source-tracking and taxonomical analyses of the maternal gut, oral and placental microbiome

**Figure 1:**
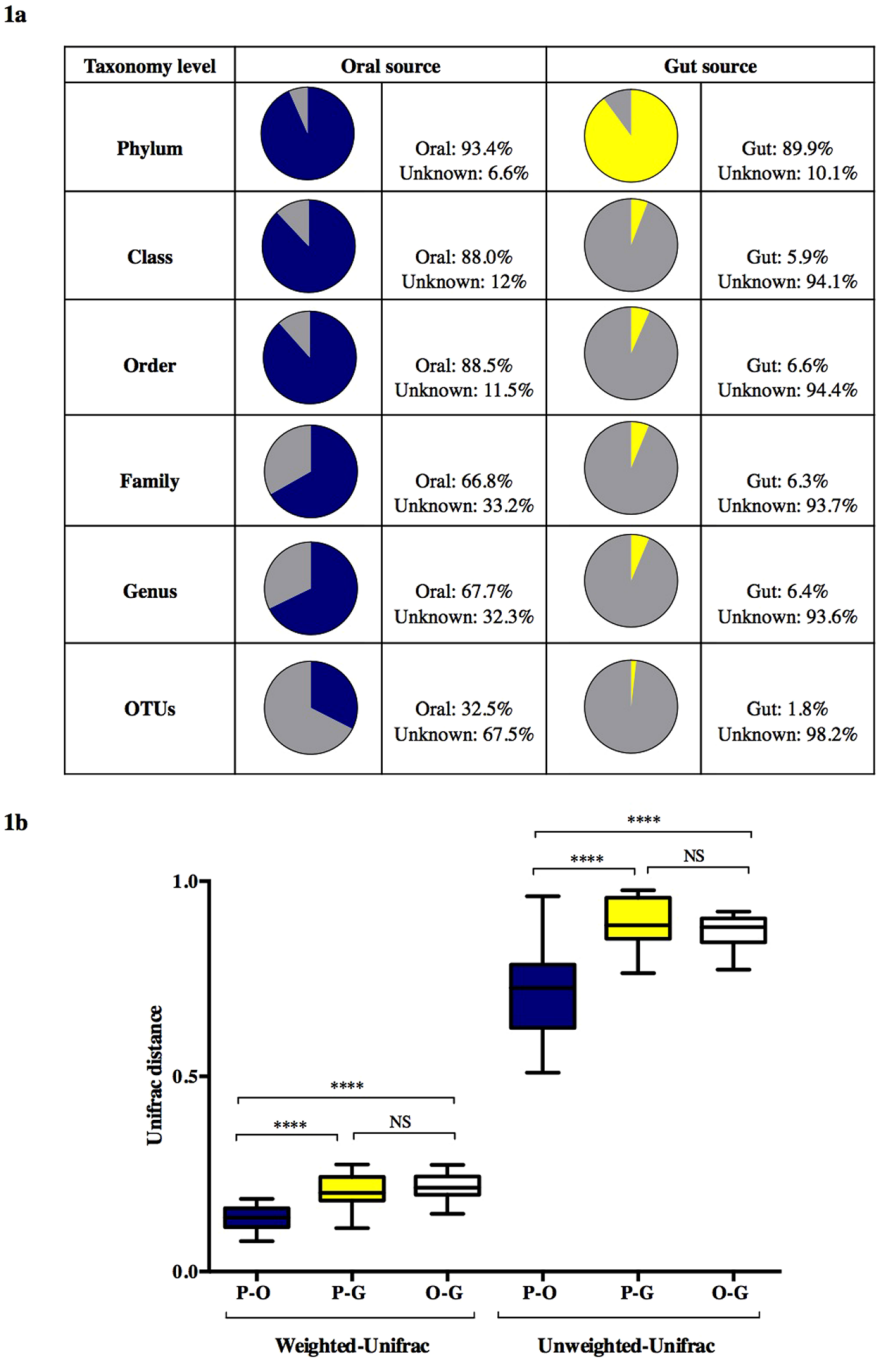
Maternal oral and gut microbial influences on the placental microbiome (**a**). Bayesian source-tracking results for placental samples at different taxonomic levels. Proportions of the maternal oral (blue), gut (yellow) and unknown source of environment (grey) on the placental microbiome. Placental samples showed a greater degree of similarity with the maternal oral microbiome. (**b**) Boxplots showing distances from unweighted and weighted Unifrac distances between the maternal oral (blue) and gut (yellow) with respect to the placental microbiome and between the oral and gut (white) microbiomes (permutations = 999). Each boxplot shows the median, lower and upper quartiles of the Unifrac distances. A lower Unifrac distance shows a greater resemblance between the two microbial communities. Pair-wise comparison were done by Mann-Whitney U tests and annotated as ****p < 0.0001 and NS: not significant.

To investigate the contribution of the maternal oral and gut microbiome to the establishment of the placental microbiome, 16S rRNA sequencing was performed in 98 samples from 37 pregnant women. The placental microbiome is relatively limited in abundance when compared to the maternal oral and gut microbiomes. On average, placental samples yielded less than a third of bacterial reads present in the maternal gut and oral microbiomes (Supplementary Figure 4). To distinguish between placental samples and contamination introduced during DNA extraction, purification and amplification, unsupervised ordination methods showed a separate clustering between pooled negative control and placental samples (R = 0.995, *p* = 0.021) (Supplementary Figure 5). Four main bacterial phyla (*Firmicutes, Bacteroidetes, Actinobacteria* and *Proteobacteria*) were identified in all microbiomes (Supplementary Figure 3). *Firmicutes* and *Proteobacteria* were highly abundant in all placental samples, representing nearly 80% of total microbial abundance. In the *Firmicutes* phylum, *Streptococcus*, *Lactobacillus* and *Veillonella* were the most predominant and *Pseudomonas*, *Haemophilus* and *Acinetobacter* dominated in the *Proteobacteria* phylum. The possible origin of the placental microbiome was determined by SourceTracker analyses at all taxonomic levels (Phylum, Class, Order, Family, Genus, and OTUs). At phylum level, each placenta shared phyla with both the maternal oral and gut microbiome (Fig. 1a). At lower taxonomic levels, the placental profile showed higher resemblance with the maternal oral than gut microbiome, however the level of similarity declined with each taxonomic level. The overlap between the placental and gut microbiomes rapidly decreases to <10% from the Class level onward (Fig. 1a).

**Figure 2:**
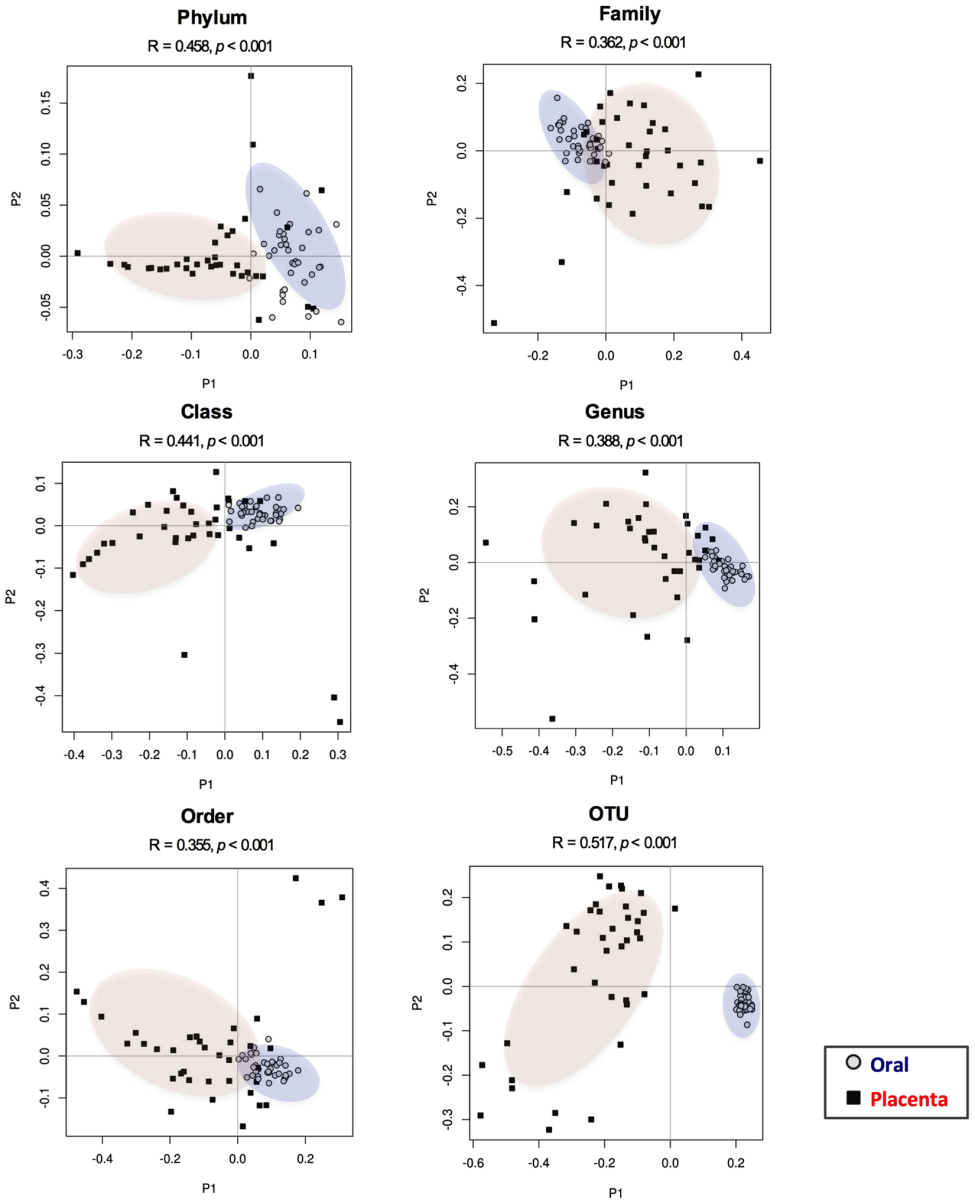
Differences in microbiome composition among the placental and maternal oral populations. PCoA plots for placental (red) and maternal oral (blue) microbiome at different taxonomic levels. Differences in microbial composition between the placenta and oral samples were determined by Anosim statistic test. An R value close to 1.0 indicates total dissimilarity between the two groups. Significant differences between the placental and oral microbiome were reported at all taxa levels (p < 0.001).

Phylogenetic distances revealed a significant proximity of the maternal oral with the placental microbiome (weighted: placenta-oral: 0.14 (0.11–0.16) vs. placental-gut: 0.20 (0.18–0.24) and unweighted: placental-oral: 0.72 (0.62–0.79) vs. placental-gut: 0.89 (0.85–0.90) (Fig. 1b). Despite these similarities between the placenta and the maternal oral microbiome, the overall microbial composition at different taxonomic levels of the placental microbiome (visualized through PCoA plots and measured by the Anosim test) showed a unique and highly variable phylogenetic clustering (*p* < 0.001) clearly separating the samples based on tissue origin (Fig. 2). PCoA plots and clustering significance of all three body sites are included in Supplementary Figure 6.

### The placental core microbiome shares phylotypes with the maternal oral and gut microbiome

**Figure 3:**
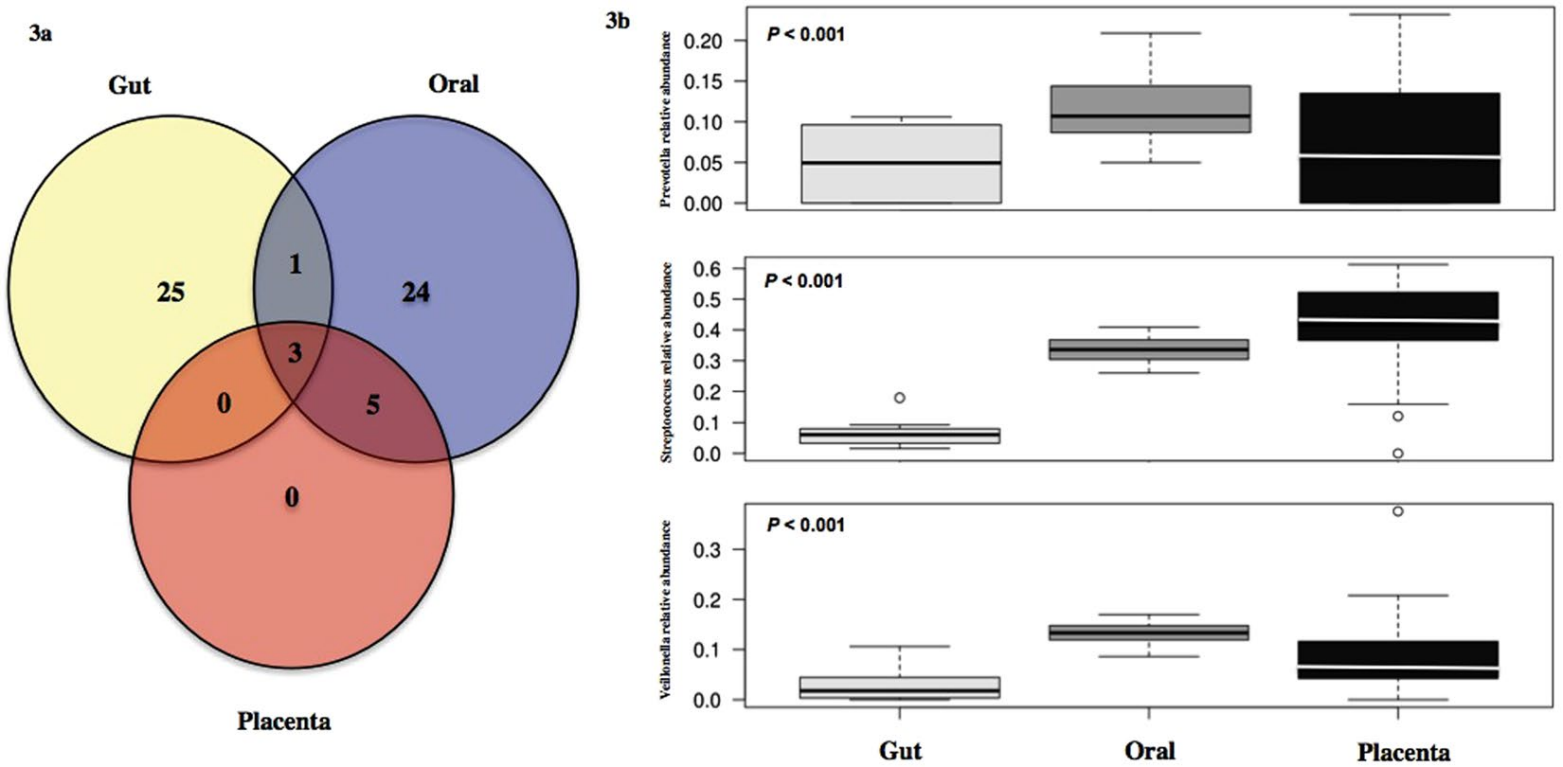
Core shared and distinct genera between the maternal gut, oral and placental microbiome. (**a**) Core microbiomes consisting of genera detected in >50% of samples from each body site were obtained and plotted in a Venn diagram. Three shared genera: *Prevotella, Streptococcus* and *Veillonella* were present in all samples. No unique core genera was detected in the placenta, all were shared between the two maternal microbiomes. Detailed genera among the three body sites are listed in Supplementary Table 3. (**b**) Relative abundances of genera: *Prevotella, Streptococcus* and *Veillonella* in all three maternal compartments. Relative abundances among body sites and genera were significantly different (p < 0.001).

The core microbiome of each compartment (as defined in the Materials and Methods) were compared. We chose to focus on what we define as a core microbiome, to provide a more conservative interpretation of the consequences of the interindividual variations we report here. The list of all phylotypes at both family and genus level between the different sample types are listed in Supplementary Figure 7 and Supplementary Table 3. All phylotypes assigned to the core placental microbiome were also present within the maternal oral and gut microbiomes. At the family- and genus-levels of classification, the placental microbiomes did possess phylotypes not detected in the other microbiomes, but these phylotypes were not part of the core placental microbiome. The proportion of families that were unique to the core microbiome of the oral cavity was 0.36 whereas it was 0.30 for the gut samples. At genus level, 41% of all genera were unique for the oral and 43% of genera unique for the gut core microbiome respectively. The core families: *Actinomycetaceae, Micrococcaceae, Oxalobacteraceae, Neisseriaceae, Pasteurellaceae* and *Pseudomonadaceae* and genera: *Actinomyces, Rothia, Haemophilus, Pseudomonas* were shared between the oral and the placental microbiomes. No families or genera were exclusively shared by the gut and the placental microbiome. All three microbiomes shared the families *Prevotellaceae, Streptococcaceae, Veillonellaceae* and *Enterobacteriaceae*, representing 8.5% of all core families (Supplementary Figure 7). The abundance of these four families in all placental samples represented 33.3% of total bacterial abundance. Similarly, at genus level, *Streptococcus, Veillonella* and *Prevotella* were shared between all body compartments (Fig. 3a). *Streptococcus* had the highest abundance (0.43 (0.18–0.62)) followed by *Veillonella* (0.07 (0.02–0.22)) and *Prevotella* (0.06 (0.01–0.26)) (Fig. 3b) in placental samples and their abundances differed significantly (*p* < 0.001) across the three different body sites. A total of 38 OTUs belonging to *Streptococcus, Veillonella* and *Prevotella* were identified in all body compartments (Supplementary Figure 8). Nine OTUs were shared between the three body sites, however only *Veillonella dispar* was taxonomically assigned to species-level.

To exclude that this effect was driven by cross contamination at delivery, for instance by fecal contamination of placental tissue, differences in the placental core microbiome between cesarean (n = 17) and vaginal delivered (n = 20) placentas were explored. No phylogenetic significant clustering was evident between cesarean vs. vaginal deliveries at either family (R = 0.019, *p* = 0.259) or genus level (R = 0.016, *p* = 0.272) nor were differences in the number of different taxa (*p* > 0.5) and richness (*p* > 0.5) present (Supplementary Figure 9). Moreover, none of the taxa (at family and genus level) mentioned previously were found to have different abundance between vaginally delivered and cesarean placentas. Detailed FDR values are listed in Supplementary Figure 10. Remarkably, no differences in family *Lactobacillaceae* (*p* = 0.20) and genus *Lactobacillus* (*p* = 0.32), which are known to be abundant in the vaginal tract, were noted between cesarean and vaginally delivered placentas.

### The placental microbiome has a distinct functional profile

**Figure 4:**
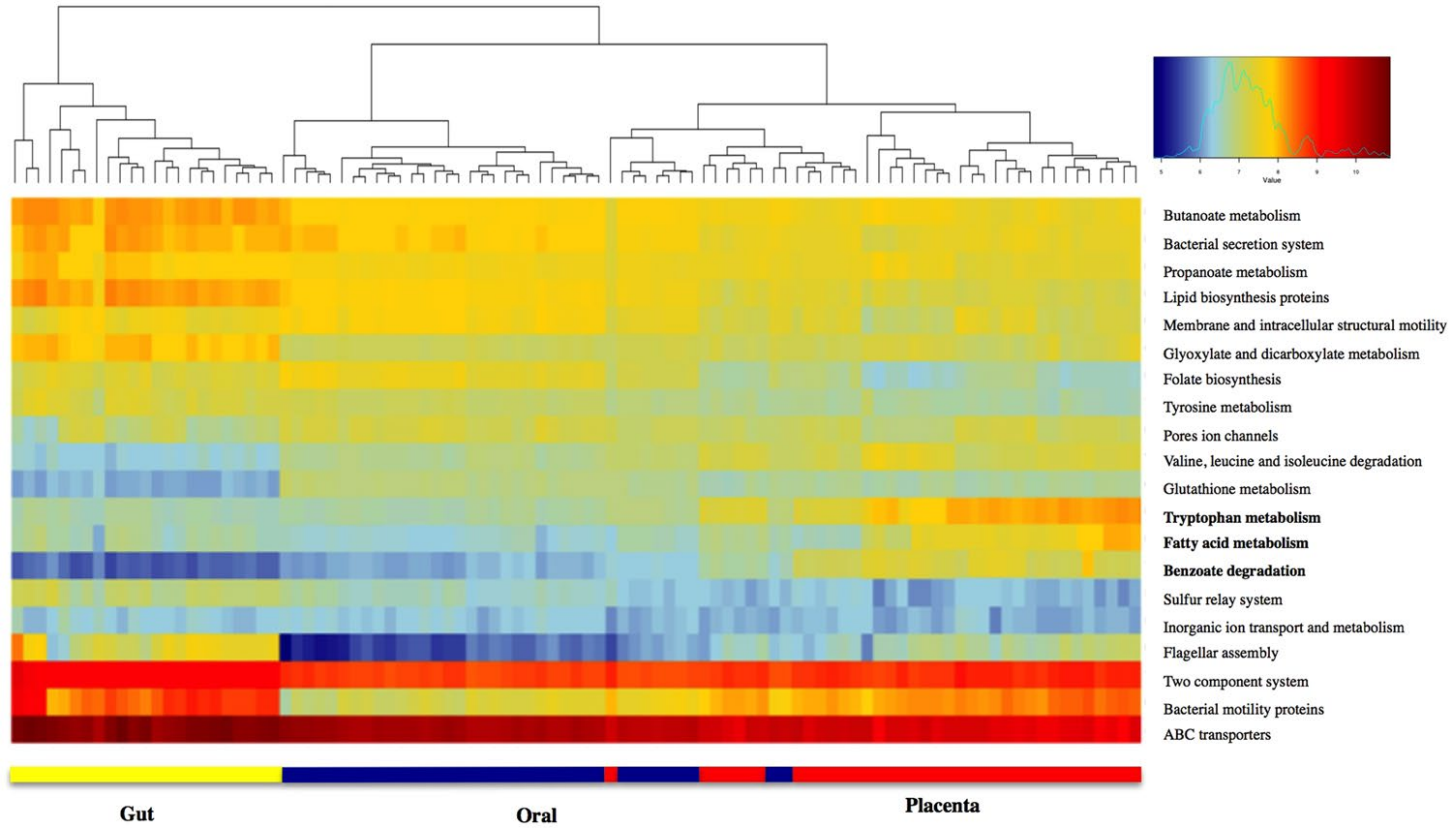
Distinct predictive metabolic profile in the placental microbiome. Heat map demonstrating the predictive functional profiling of microbial communities in the placenta, gut and oral samples, using 16s rRNA gene sequences. Stronger intensity of red indicates higher pathway activity and blue lower activity. Significant microbial functional pathways determined by the LEfSe algorithm are displayed in this heat map (LDA score > 3.0). Bolded pathways were significantly enriched in the placenta in comparison to the maternal oral and gut microbiome.

Using PICRUSt, the predicted core functions of the placental microbiome were compared to those of the core maternal oral and gut microbiome. Pathways encoding for carbohydrate metabolism, bacterial structure and vitamin-related pathways were overrepresented in the oral and gut microbiome (Fig. 4). In contrast, microbial communities in the placenta appear to be enriched with genes related to tryptophan, fatty acid metabolism and benzoate degradation (Fig. 4 and Supplementary Figure 11 (LEfSe analysis)).

## Discussion

The purpose of this study was to investigate possible seeding sources for the bacterial DNA detected in the placenta, which is currently unclear. Similarities between the oral non-pregnant and placental microbiome have been previously suggested^10^. Distinct functional profiles and microbial populations are detected in the placental microbiome. The present study is the first to show that the placental bacterial profile is most similar to the pregnant oral microbiome and less alike the maternal gut microbiome in the same individual. However, the core microbiomes of the three body sites share some distinct taxa.

Thirty-seven term placentas of overweight and obese pregnancies not complicated by preterm birth, GDM or preeclampsia were selected for this substudy, as these factors are associated with an altered placental microbiome composition^12–15^. All placentas displayed the presence of low abundance of non-pathogenic bacteria. Comparable results have previously been reported^10, 12, 14^. However, a recent study reported no differences between placental samples and contamination controls^16^. Based on placental samples obtained from 6 uncomplicated pregnancies, only a small number of sequences was detected in placental samples and no clear clustering was observed between samples and environmental and reagent controls. In contrast, the 37 placental samples in this study yielded on average 4.4 times more sequences than the negative control and show a clear clustering that is distinct from the pooled reagent and PCR negative controls. The differences between the studies may be a result of the use of different regions of the 16S rRNA gene. Lauder *et al*. used V1-V2 primers, which may have a predilection for environmental and ecological niche contamination and thus biased their study towards detecting contaminants rather than non-contaminants. Here, the V6–V8 region of the 16S rRNA gene was targeted, covering a slightly larger amplicon size. However the main taxa detected in contamination controls by Lauder *et al*.^16^ including *Bradyrhizobiaceae, Methylobacterium, Comamonadaceae, Propionibacterium* and *Sphingobacterium* were also detected in the negative controls of this study. These sequences may reflect contamination of reagents in the kits used for extraction and sequence generation.

This study is unique in determining the possible origins of the placental microbiome by comparing it with the oral and gut microbiomes at all taxonomic levels within the same individual. The placental microbiome shows the greatest resemblance to the oral microbiome especially at high taxonomic levels (e.g. phylum, class, order) but the overlap decreases significantly at lower taxonomic levels. Aagaard *et al*.^10^ reported a higher similarity of the placental microbiome with the oral microbiome from unrelated non-pregnant subjects from the US human microbiome project (HMP), although comparisons were only reported at phylum level. Phylogenetic analyses showed that the placental microbiome clusters independently from the maternal oral microbiome at all taxonomic levels emphasizing a distinct placental microbial community. This could be a result of the tissue-specific environmental conditions that enable the colonization by specific bacteria belonging to the same higher taxonomic category *e.g*. different families belonging to the order or class of bacteria.

Due to the interindividual variability of bacterial communities, the core microbiome for all three environments was used to assess the potential source for the placental microbiome. Analyses at family and genus levels show a greater share of phylotypes between the maternal oral and placenta microbiomes. Interestingly, four families and three genera belonging to the same families are shared between all three maternal microbiomes: *Veillonella, Streptococcus* and *Prevotella*, are commonly present in placenta, oral and gut microbiomes^10, 17–19^. These genera are frequently observed in oral and gut microbiome samples^20, 21^, especially in healthy subjects, with all genera detected in both the oral and stool microbiomes of ~45% of the subjects examined as part of the US Human Microbiome Project (HMP)^22^. It has also been proposed that the gastric cavity possesses a core microbiome, comprised of phylotypes assigned to *Prevotella, Streptococcus, Veillonella, Rothia* and *Haemophilus* ^23^. Specific oral microorganisms can seed distal sites below the stomach, which may explain the abundance in the pregnant women’s digestive tract and the selective enrichment of these genera in the placenta. These genera were also found in most of the placental samples examined in this study. In pregnancy, the gut barrier has increased leakiness^24^ and pregnant women have increased prevalence of bleeding in the oral cavity^25^. These changes are associated with the hormonal and cardiovascular changes of pregnancy. It is therefore possible for *Prevotella*, *Streptococcus* and *Veillonella* to enter the bloodstream from the oral cavity and/or the gut lumen to colonize the placenta. In addition, these genera are successful colonizers and biofilm producers^26–28^, facilitating their ability to thrive within many different body sites.

In a mouse model of periodontal infections, injection of bacteria-including *Veillonella, Streptococcus* and *Prevotella* spp—obtained from human saliva and subgingival plaques-- into the tail vein causes placental translocation of these bacteria^8^. However, not all bacteria present in the pooled oral samples were translocated to the murine placenta, suggesting once more that the nutritional and physiological ecologies of the placenta imposes selective pressures affecting microbial colonization and persistence. In that context, we identified *Veillonella dispar* as a common species between the three body sites. This species has been found in neonates, is associated with an inherent maternal gut microbiome and persists at least throughout the first year of life^29^. For growth, it utilizes short-chain organic acids, particularly lactate^30^. Lactate is produced by the uteroplacental tissues, where it is used for fetal energy production^31^. Thus, the placenta may be an optimal niche for *V. dispar* and could possibly have a cooperative metabolic system with the placenta. Recent studies have also revealed by co-culturing of isolated intestinal *Streptococcus* and *Veillonella* strains that these strains have combined immunomodulatory properties that differ from those of the individual strains^32^. The presence of these genera in the placenta may therefore trigger immunomodulatory mechanisms that may influence placental immune homeostasis.

Even though key placental phylotypes are shared with the maternal oral and gut microbiomes, there is evidence for a unique placental microbiome. The results of this study suggest that the placental microbiome does not have only one specific and unique source of colonization but that selective bacteria translocated from different maternal microbiomes could contribute to the assemblage of the placental microbiome. Even though murine models have well documented the transmission of oral microorganisms in the placenta^8, 9, 33, 34^, increased gut bacterial translocation during pregnancy and lactation has been also been reported^7^. Maternal gut microorganisms are transported to mesenteric lymph nodes and mammary gland by mononuclear cells during late pregnancy and lactation in mice^7^. Moreover, specifically labeled intestinal bacteria have been recovered from murine placentas^35^. The results of this study indicate that bacteria from the maternal oral cavity and gut could employ specific translocation mechanisms to colonize the placenta.

Interestingly, the bacteria that make up the placental microbiome are predicted to have distinctive functional capacities relative to the other microbiomes. While the role of oral and gut microbes is related to carbohydrate and amino acid metabolism, as well as vitamin biosynthesis^36, 37^, the placental microbiomes are predicted to be enriched with genes regulating tryptophan, fatty acid metabolism and benzoate degradation. Placental tryptophan metabolism is important for neurodevelopment in the fetus and perturbations of placental tryptophan metabolism have been associated with altered neurodevelopmental processes in the fetus^38^. Catabolism of tryptophan in the placenta is linked with the establishment and maintenance of the feto-maternal immune tolerance^39^, placental circulation and growth^40^ and modulation of antimicrobial activity^41^ by inhibiting ascending infections from the vagina^39^. This overrepresented pathway in placental microbes may indicate a selective mechanism of natural placental colonizers to prevent colonization by foreign microorganisms. *Veillonella* species are equipped with the beta subunit responsible for the synthesis of L-tryptophan, possibly explaining the enrichment of tryptophan metabolism pathway in our dataset. Pathways encoding fatty acid metabolism were also enriched in placental bacteria. The placental microbiota may aid in efficient extraction of energy from circulating fatty acids and play a crucial role in supplying energy-yielding substrates to the fetus. Moreover, bacterial genes mapping to pathways involved in benzoate degradation were also enriched. Aromatic compounds such as benzoate are used as a carbon source for many microorganisms. Benzoate is commonly used as food preservative in many products including in carbonated beverages^42^. It crosses the placenta and elevated serum concentrations of benzoate have been associated with neurologic disturbances^43^. We also emphasize that our considerations here are based on a predictive analysis, and await confirmation using shotgun metagenome sequencing or other more functional approaches, coupled perhaps with dietary analysis.

The absence of vaginal swabs is a limitation of this study and restricts the analysis of the placental microbial colonization pattern only to oral and gut microbes. In European ethnic groups, the vaginal microbiome is enriched in *Lactobacillus* spp.; and while members of this genus can be readily found in the gut microbiome none were unique for the placental core microbiome. This might indicate that if the vaginal microbiome contributes to the seeding of the placental microbiome, it is not with bacteria that are unique to the vaginal microbiome. To that end, *Veillonella* spp. were essentially absent from the HMP vaginal samples^19, 22^, suggesting that the origin of these phylotypes in our placental samples were unlikely to have originated from the vagina. Furthermore, analysis of the placental microbiome of vaginal deliveries compared with those delivered by Cesarean section, showed no significant differences on microbial diversity and taxa. Taken together, we conclude our results are true representations of the placental microbiome and are not affected by contaminations of the placenta by the vaginal microbiome or maternal feces during the delivery process. The sequencing strategy was based on the 16S rRNA gene amplification restraining the identification of some taxa at species level. This explains the reason why most microbial associations were based on genus level and metagenomic pathways were only inferred. In addition, the disappearance of overlap between the placenta and other maternal sites from Class level and below might be due to primer sets used and depth of sequencing. The phyla and taxa reported in this study parallels that of Aagaard *et al*.^10^ using whole genome shotgun sequencing but are fairly different from that of Collado *et al*.^5^. Different regions of the 16S rRNA gene can return different results and therefore studies might not be directly comparable. The use of whole genome sequencing provides better resolution and reduces amplicon bias. This study cannot provide evidence whether the bacteria detected in all placental samples are alive and metabolically active. In addition, appropriate environmental controls would be beneficial to exclude all potential contamination in the present placental samples. Moreover, as the gut microbiome undergoes structural changes throughout pregnancy becoming less diverse in each individual but more variable between individuals, fecal samples obtained during the third trimester may provide further insights into the shared placental-gut phylotypes. The use of core microbiomes including only those bacteria present in ≥50% of participants reduces the risk for over interpretation of consequences of interindividual variation. And since it is not yet clear when in pregnancy the placental microbiome is seeded, comparisons with the gut microbiome in early second trimester may even be more appropriate.

## Conclusion

This study provides evidence that both the maternal oral and gut microbiome may contribute to the seeding of the placental microbiome. The placental microbial communities show a higher similarity with the bacteria found in the maternal oral microbiome than the gut microbiome especially at higher taxonomic levels. The presence of shared phylotypes between all three compartments suggests that placental bacteria sequences may have multiple niche sources. Given that the gut microbiome undergoes structural changes throughout pregnancy^44^ becoming less diverse in each individual but more variable between individuals, stool samples obtained during the third trimester may provide further insights into shared placental-gut phylotypes. However, since it is not yet clear when in pregnancy the placental microbiome is seeded, comparisons with the gut microbiome in early second trimester are important and may even be more appropriate. Further studies into the significance of intrauterine microbial populations and dynamics could identify their role in feto-placental communication, fetal development and health.

## Materials and Methods

### Ethics, consent and permission

This study was approved by the Human Research Ethics committees of the Royal Brisbane and Women’s Hospital (HREC/RBWH/11/467) and The University of Queensland (2012000080). Written informed consent was obtained from all participants prior to enrolment in the trial. All experiments were performed in accordance with relevant guidelines and regulations.

### Study subjects and sample collection

Overweight and obese pregnant women included in this study were participants in the double-blind randomized controlled trial: SPRING (Study of Probiotics IN the prevention of Gestational diabetes) (ANZCTR 12611001208998)^11^. In total 37 women were selected for having a complete matched sample set of placenta (n = 37) and maternal oral (n = 37) with 24 mothers also having maternal fecal samples available. Detailed clinical characteristics of these women are presented in Table 1. Maternal feces, oral swabs and placental samples were collected at separate times antepartum and postpartum: Maternal fecal samples were self-collected at 16 weeks gestation, refrigerated and stored at −80 °C within 24 hours of collection. At 36 weeks gestation, maternal oral swabs were collected by sterile dry swab (Copan Diagnostics, Murrieta, CA) and stored immediately at −20 °C prior to transfer to −80 °C. Term placentas were collected by clinical practitioners. Within 1 hour of delivery each placenta was transported to the laboratory for processing. Trained researchers were provided with sterile supplies and stringent instructions to excise cuboidal 1 cm^3^ sections from the fetal side of the placenta. Excisions were placed in autoclaved 2 mL tubes and immediately placed in liquid nitrogen and stored at −80 °C until processing.

### Sample processing and microbiome sequencing

Genomic DNA was isolated from maternal feces, placental tissue and oral swabs. A total of 0.25 grams of stool sample was extracted using the repeated bead beating and column (RBB + C) method followed by the Qiagen AllPrep DNA extraction kit as previously detailed^45, 46^. DNA extraction and recovery from oral and placental tissue (10 mg) was achieved by the automated Maxwell 16 system following mechanical disruption using sterile zirconia beads (0.1 and 0.05 mm diameter) in 300 μL lysis buffer (NaCl 0.5 mol/L, Tris–HCl 50 mmol/L, pH 8.0, EDTA 50 mmol/L and SDS 4% w/v). The Maxwell 16 Buccal Swab LEV DNA Purification kit and Maxwell 16 Tissue DNA Purification kit (Promega, Madison, WI, USA) were used following the manufacturer’s recommendations. Negative controls consisting of extraction reagents and PCR amplification were added to each set of fecal, oral and placental samples and included in the sequencing reaction. Purified DNA was quantified by the Nanodrop ND 1000 spectrophotometer (Nanodrop Technologies). Oral, fecal and placental DNA extractions were performed separately to avoid cross contamination between samples.

A barcoded primer set based on universal primers 926F (5′-TCG TCG GCA GCG TCA GAT GTG TAT AAG AGA CAG AAA CTY AAA KGA ATT GRC GG -3′) and 1392R (5′ -GTC TCG TGG GCT CGG AGA TGT GTA TAA GAG ACA GAC GGG CGG TGW GTR C-3′) was used to amplify 500 bps of the hypervariable V6–V8 region of the 16S rRNA gene. Specificity and amplicon size were verified by gel electrophoresis. PCR products were cleaned with AMPure XP beads. Amplicons were barcoded using the Nextera XT Index Kit set A and set B. Amplicons were purified with the Promega Wizard Gel Extraction kit, followed by a second AMPure XP beads cleaning to reduce potential contamination with human DNA. Fecal, oral and placental tagged-amplicons were quantified, normalized and pooled. The pooled libraries were sequenced using the Illumina MiSeq platform and workflows established at the University of Queensland’s Australian Center for Ecogenomics (www.ecogenomic.org). Sequences were deposited in the NCBI database (SUB 2559729). Sequences were joined, demultiplexed and quality filtered using QIIME (Quantitative Insights Into Microbial Ecology v 1.9.1)^47^. An open reference OTU picking method using 97% identity to the Greengenes 13_8 database^48^ was selected. OTUs with a relative frequency below 0.01 were removed. Resultant data demonstrated a total median sequenced reads of 44259.5 (IQR: 38477–53917), 76707 (IQR: 58486–125071) and 60478 (IQR: 13965–121911) for fecal, oral and placental samples respectively. A total of 34, 38 and 26 different genera were identified in fecal, oral and placental samples respectively. The alpha diversity curve for all the sample types determined by Chao 1 is provided in Supplementary Figure 1.To evaluate the potential impact of contamination in each set of samples, a set of reagent controls, to which no additional tissue or DNA was added were included. A DNA extraction control and PCR amplification control were processed in an identical manner to the rest of the samples, starting from the lysis step. From each set of samples, the DNA extraction control and the PCR amplification control were pooled together and sent for sequencing. Quality filtering and OTUs detected in the negative controls were deleted from the generated OTU tables (Supplementary Table 1). Pooled placental negative controls yielded 13840 sequenced reads. The OTUs detected in negative controls are listed in Supplementary Table 2, representing 13 different genera. To examine if the sequence reads in the negative control were distinct from those reported in placental samples, unsupervised ordination methods (PCoA, PCA and NMDS) were used to identify placental and pooled negative control clustering. Due to the different origin of the samples, OTU tables at each taxonomy level (phylum, class, order, family, genus and OTUs) were normalized to relative abundance using the cumulative sum scaling (CSS) normalization method after deletion of the sequences present in the negative samples^49^. Relative abundances at phylum level after and before normalization are reported in Supplementary Figures 2 and 3.

### Microbial diversity and statistical analysis

To investigate the seeding role of the maternal oral and gut microbiome to the placental microbiome, bacterial source tracking analyses were performed using SourceTracker v 1.1^50^. Oral and gut samples were designated as sources and the placental sample of the corresponding mother was selected as sink. Alpha and beta diversity were calculated on normalized OTUs tables. Alpha diversity was measured by the Chao 1 and Shannon indices, representing the number, richness and distribution of taxa. Beta diversity was calculated using both phylogenetic (Unifrac distance) and non-phylogenetic (Bray-Curtis) distances matrices and visualized through PCoA. Anosim testing was used to confirm significant differences in microbial community composition. Microbial diversity analyses were performed within QIIME and with the Calypso software tool (http://bioinfo.qimr.edu.au/calypso/). Core microbiomes consisting of OTUs detected in 50% of samples from each dataset were obtained. Placental, gut and oral phylotypes at genus and family level were plotted using Venny 2.1^51^. Clinical metadata is presented as median with interquartile range in all instances (Table 1).

### In silico metagenomics using PICRUSt

Functionality of the different metagenomes, grouped by oral, gut and placenta were predicted using the software PICRUSt 1.1.0^52^. This tool predicts the functional composition from the 16S rRNA gene data based on the Kyoto Encyclopedia of Genes and Genomes (KEGG) Orthologs classification. Significant differences in microbial functional pathways were tested by the LEfSe (Linear Discriminant Analysis Effect Size) algorithm (http://huttenhower.sph.harvard.edu)^53^.

### Data availability statement

Sequences were deposited in the NCBI database (SUB 2559729).

## Electronic supplementary material

Supplementary information
