# Supplementary material for: Contributions of the maternal oral and gut microbiome to placental microbial colonization in overweight and obese pregnant women
Source: Sci Rep. 2017 Jun 6;7:2860. doi: 10.1038/s41598-017-03066-4 (PMC5460277; doi:10.1038/s41598-017-03066-4)

<u>Supplementary Information</u>

Contributions of the maternal oral and gut microbiome to placental microbial colonization in overweight and obese pregnant women.

Luisa F. Gomez-Arango, Helen. L. Barrett, H. David. McIntyre, Leonie K. Callaway, Mark Morrison, Marloes Dekker Nitert.

Supplementary Table 1. Total number of OTUs before and after quality filtering.

Body site	Total number of OTUs before quality filtering	Total number of OTUs after quality filtering
Fecal	645589	644579
Oral	2154107	2074522
Placenta	29765	26972

Supplementary Figure 1. Rarefaction analysis by Chao 1 Index showing sequencing data from oral (blue), fecal (red) and placenta (orange).

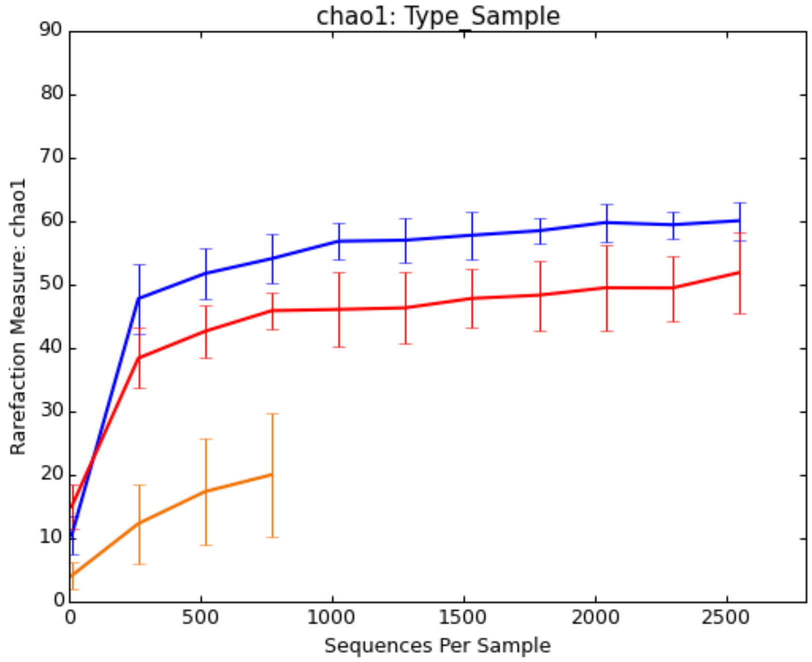

Supplementary Table 2. List of OTUs detected in the placental negative controls.

Family	Genus	Species	Number of identified taxa
Bradyrhizobiaceae	*Afipia*		1
Dermabacteraceae	*Brachybacterium*	*conglomeratum*	2
Bradyrhizobiaceae			4
Bradyrhizobiaceae	*Bradyrhizobium*	*elkanii*	2
Comamonadaceae			2
Corynebacteriaceae	*Corynebacterium*		6
Aerococcaceae	*Facklamia*		1
Lactobacillaceae	*Lactobacillus*		2
Lactobacillaceae	*Lactobacillus*	*salivarus*	2
Methylobacteriaceae	*Methylobacterium*		3
Norcardioidaceae			2
Propionibacteriaceae	*Propionibacterium*	*acnes*	1
Pseudoalteromonadaceae	*Pseudoalteromonas*		1
Pseudomonadaceae	*Pseudomonas*		3
Rhodobacteraceae	*Rhodobacter*		1
Rhodobacteraceae			1
Rhodocyclaceae			1
Sphingobacteriaceae	*Sphingobacterium*	*multivorum*	2
Staphylococcaceae	*Staphylococcus*		9
Staphylococcaceae	*Staphylococcus*	*aureus*	2
Staphylococcaceae	*Staphylococcus*	*epidermis*	4
Streptococcaceae	*Streptococcus*		2

Supplementary Figure 2. Differences in the relative abundance of gut, oral and placental taxa at phylum level before normalization.

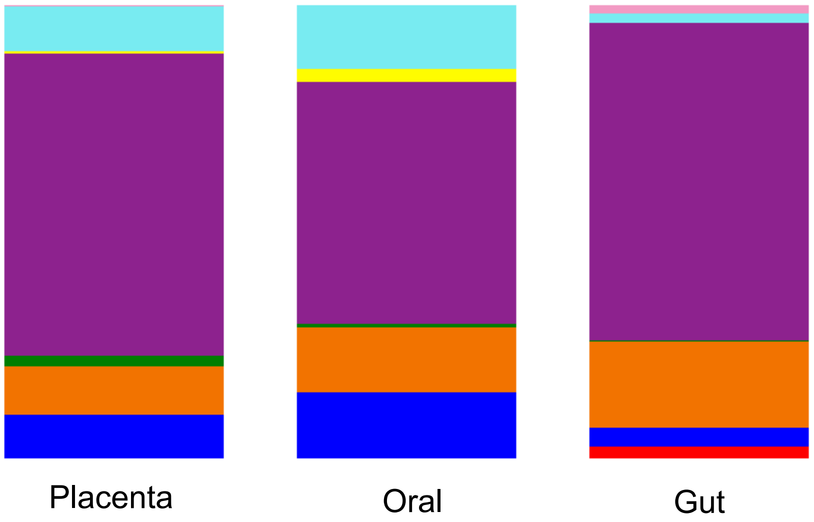

Taxonomy	Gut	Oral	Placenta
Euryarchaeota	1.1%	0%	0%
Actinobacteria	2.6%	14.1%	9.7%
Bacteroidetes	15.8%	14.2%	10.6%
Cyanobacteria	0.1%	0.9%	2.5%
Firmicutes	77.8%	53.3%	66.3%
Fusobacteria	0%	2.8%	0.4%
Proteobacteria	1.5%	14.0%	9.9%
Spirochaetes	0%	0.3%	0.2%
Synergistetes	0%	0.1%	0%
TM7	0%	0.3%	0%
Tenericutes	0.2%	0%	0%
Verrucomicobia	0.9%	0%	0.3%
Thermi	0%	0%	0.1%

Supplementary Figure 3. Differences in the relative abundance of gut, oral and placental taxa at phylum level after normalization.

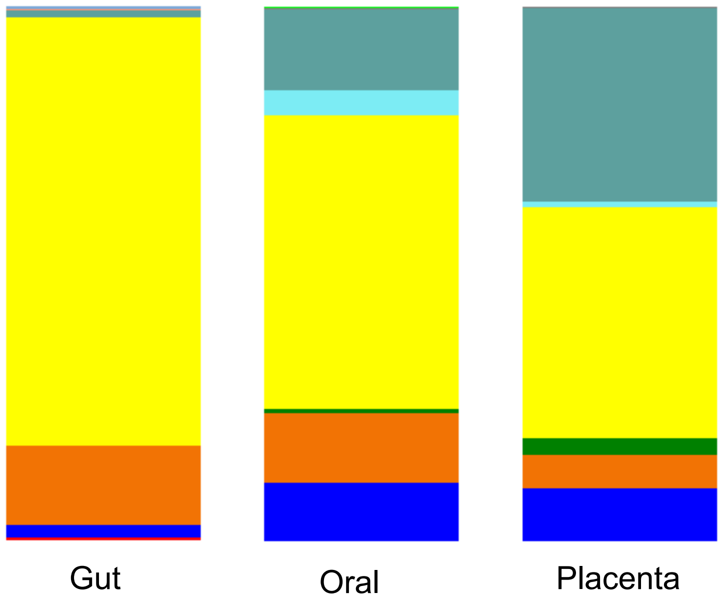

Taxonomy	Gut	Oral	Placenta
Euryarchaeota	0.5	0	0
Actinobacteria	2.3	11	9.8
Bacteroidetes	14.8	13.1	6.3
Cyanobacteria	0	0.6	3.1
Firmicutes	80.3	55.1	43.5
Fusobacteria	0	4.6	0.9
Proteobacteria	1.4	15.0	36.3
Spirochaetes	0	0.2	0.1
Synergistetes	0	0.1	0
TM7	0	0.3	0
Tenericutes	0.2	0	0
Verrucomicobia	0.5	0	0
Thermi	0	0	0.1

Supplementary Figure 4. Differences in normalized reads distribution between the gut, oral and placental samples after.

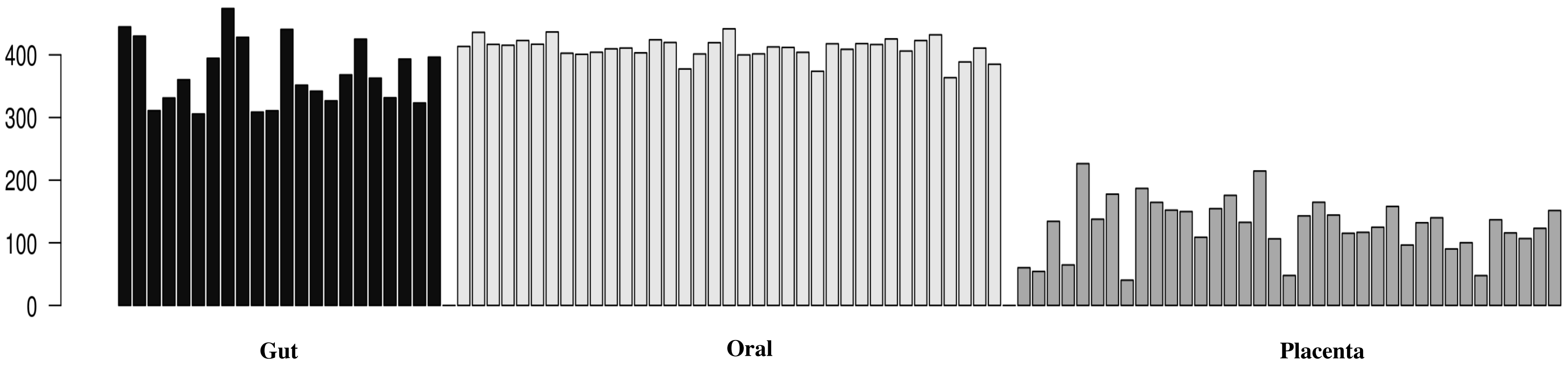

Supplementary Figure 5. Clustering of placental samples and negative control using unsupervised ordination methods. S2a) Non-metric multidimensional scaling analysis based on the pairwise dissimilarity between the placenta and negative control samples. **S2b)** Principal component analysis showing the variance between placental samples and negative control. **S2c)** Principal coordinate analysis showing the phylogenetic distance between placental samples and negative control.

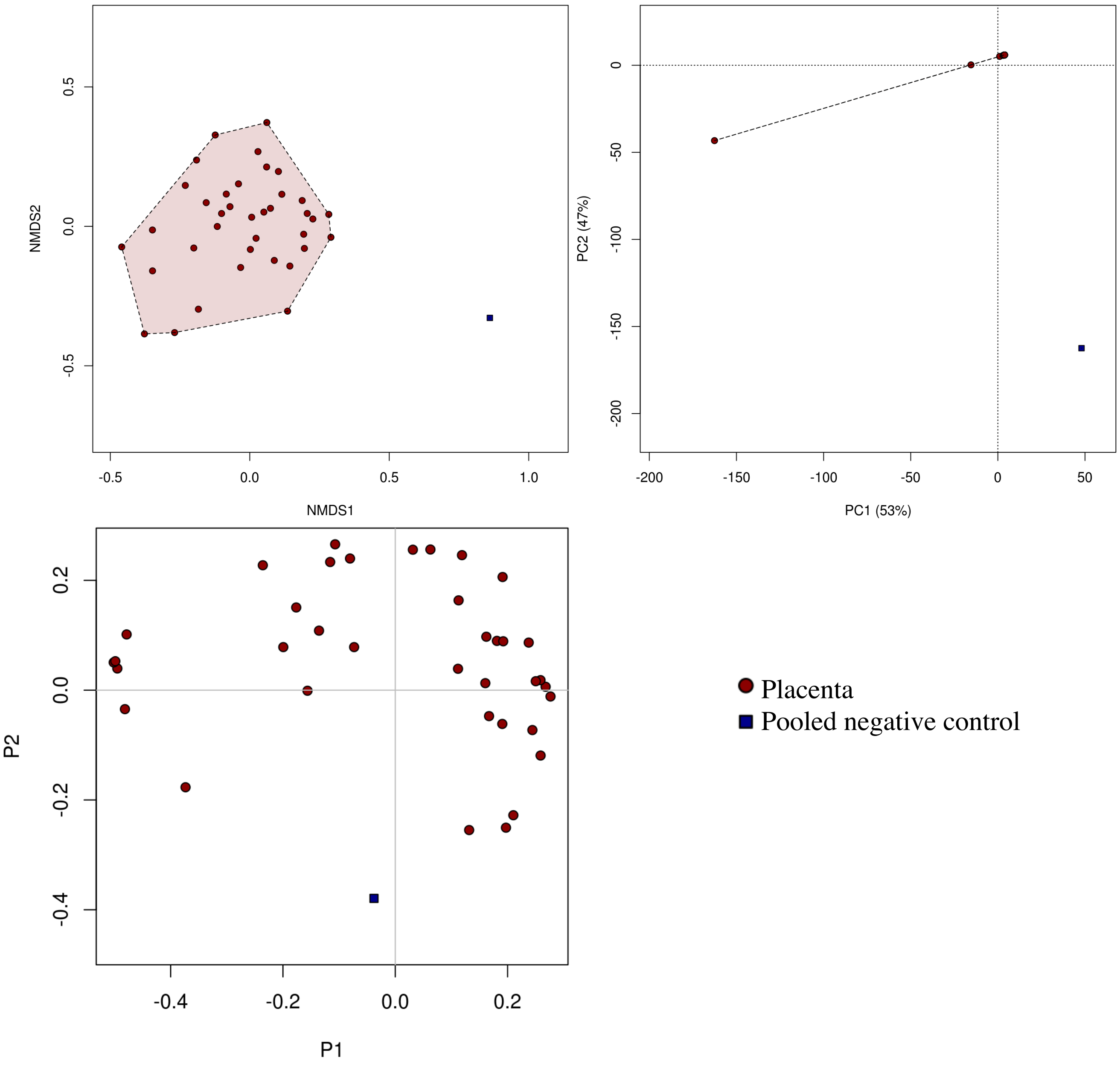

Supplementary Figure 6. Differences in microbiome composition among the gut, oral and placental samples.

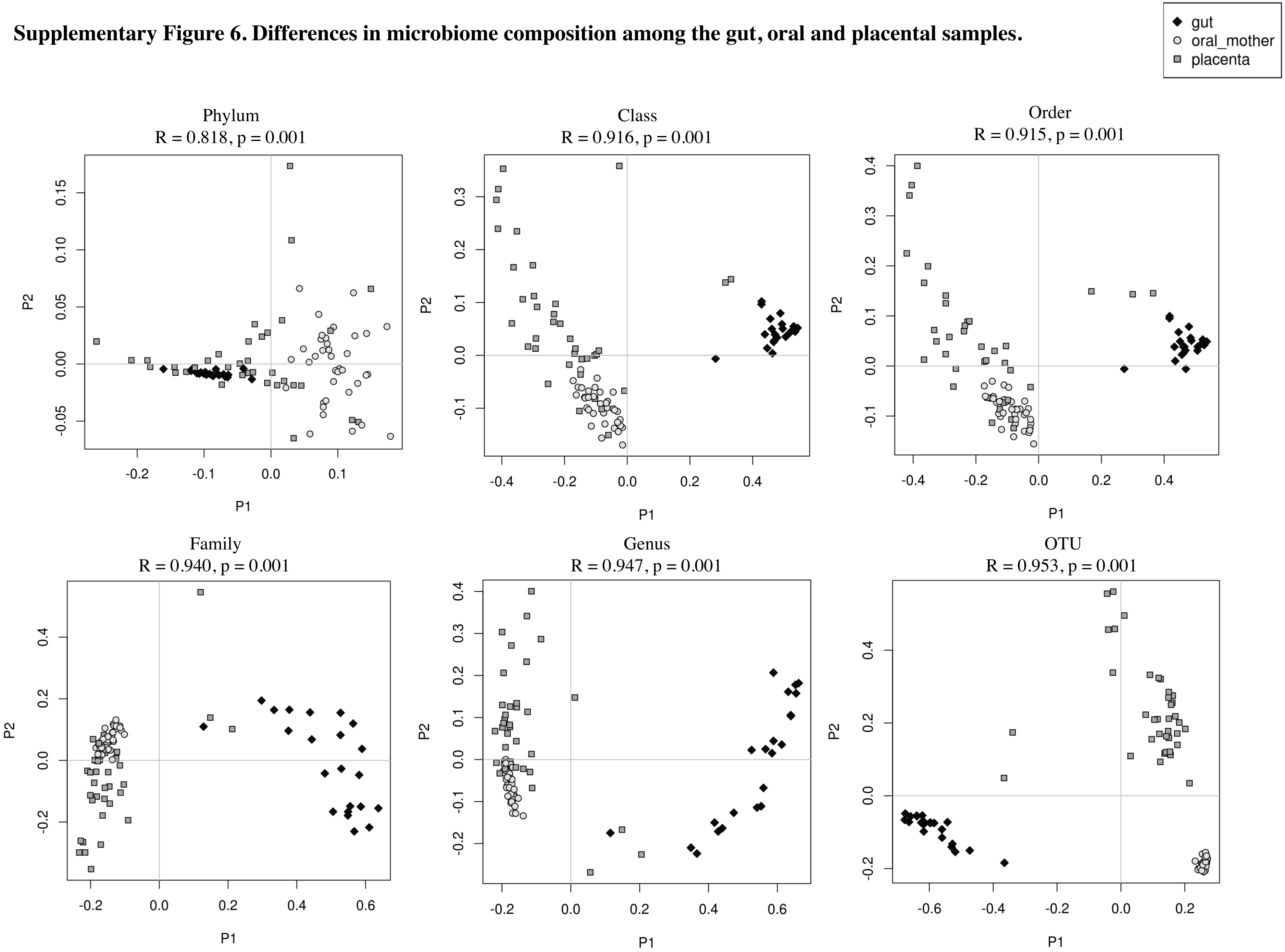

Supplementary Figure 7. Core shared and distinct families between the maternal gut, oral and placental microbiome. All families present in the core microbiome of each body site are listed.

Core Gut

Methanobacteriaceae
Bacteroidaceae
Rikenellaceae
Barnesiellaceae
Odoribacteraceae
Bacillaceae
Christensenellaceae
Clostridiaceae
Ruminococcaceae
Mogibacteriaceae
Erysipelotrichaceae
Alcaligenaceae
Desulfovibrionaceae
Verrucomicrobiaceae

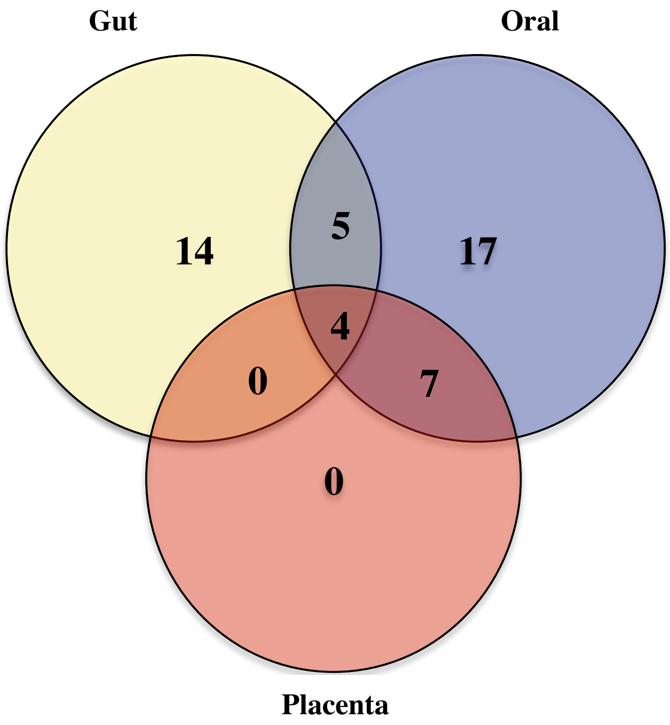

Core Oral

Corynebacteriaceae
Paraprevotellaceae
Gemellaceae
Aerococcaceae
Carnobacteriaceae
Enterococcaceae
Tissierellaceae
Fusobacteriaceae
Leptotrichiaceae
Bradyrhizobiaceae
Methylobacteriaceae
Sphingomonadaceae
Burkholderiaceae
Comamonadaceae
Aeromonadaceae
Spirochaetaceae
Rs-045

Core Gut-Oral

Bifidobacteriaceae
Coriobacteriaceae
Porphyromonadaceae
Lachnospiraceae
Peptostreptococcaceae

Core Gut-Oral-Placenta

Prevotellaceae
Streptococcaceae
Veillonellaceae
Enterobacteriaceae

Core Oral-Placenta

Actinomycetaceae
Micrococcaceae
Lactobacillaceae
Oxalobacteraceae
Neisseriaceae
Pasteurellaceae
Pseudomonadaceae

Supplementary Table 3. List of core shared and distinct genera between the maternal gut, oral and placental microbiome.

Core gut	Core oral	Core gut and core oral	Core oral and core placenta	Core oral, core placenta and core gut
Methanobrevibacter	*Corynebacterium*	*Dialister*	*Actinomyces*	*Streptococcus*
Bifidobacterium	*Scardovia*		*Rothia*	*Veillonella*
Adlercreutzia	*Atopobium*		*Lactobacillus*	*Prevotella*
Collinsella	*Porphyromonas*		*Haemophilus*	
Eggerthella	*Gemella*		*Pseudomonas*	
Bacteroides	*Aerococcus*			
Parabacteroides	*Granulicatella*			
Butyricimonas	*Catonella*			
Odoribacter	*Oribacterium*			
Clostridium	*Peptostreptococcus*			
SMB53	*Megasphaera*			
Blautia	*Selenomonas*			
Coprococcus	*Parvimonas*			
Dorea	*Fusobacterium*			
Lachnospira	*Leptotrichia*			
Roseburia	*Bradyrhizobium*			
Ruminococcus	*Methylobacterium*			
Anaerotruncus	*Sphingomonas*			
Faecalibacterium	*Lautropia*			
Oscillospira	*Kingella*			
Phascolarctobacterium	*Neisseria*			
Eubacterium	*Actinobacillus*			
Sutterella	*Aggregatibacter*			
Bilophila	*Treponema*			
Akkermansia				

Supplementary Figure 8. OTUs belonging to genera *Streptococcus*, *Prevotella* and *Veillonella* in all three sample types.

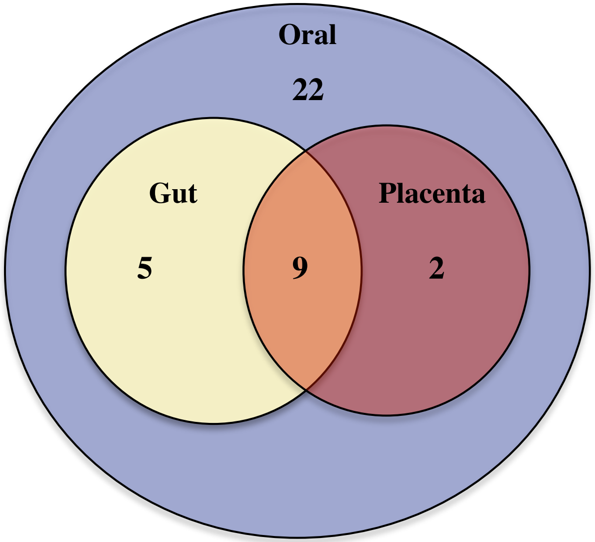

Taxa	type	details	gut.abu	oral_mother.abu	placenta.abu	gut.occ	oral_mother.occ	placenta.occ
X994939_Streptococcus__s__	pan	oral_mother&placenta	0.09	8.558	3.236	0.05	1	0.46
X937248_Veillonella__s__dispar	core	gut&oral_mother&placenta	2.185	9.997	3.655	0.55	1	0.54
X935742_Prevotella__s__melaninogenica	unique	oral_mother	0	2.853	0.173	0	0.78	0.03
X916151_Veillonella__s__	unique	oral_mother	0	3.847	0.329	0	0.7	0.05
X878104_Veillonella__s__dispar	pan	gut&oral_mother	1.983	7.445	1.597	0.55	1	0.24
X873174_Veillonella__s__dispar	pan	gut&oral_mother	1.868	6.73	1.805	0.5	1	0.27
X861780_Prevotella__s__	unique	oral_mother	0	3.183	0.477	0	0.68	0.08
X72112_Prevotella__s__intermedia	unique	oral_mother	0	2.354	1.304	0	0.51	0.19
X593803_Streptococcus__s__	unique	oral_mother	0.309	6.845	0.901	0.18	1	0.14
X567427_k_Streptococcus__s__	pan	gut&oral_mother	1.017	3.271	0	0.5	0.76	0
X535359_Prevotella__s__	core	gut&oral_mother&placenta	1.475	7.475	2.77	0.41	0.97	0.46
X511378_Veillonella__NA	unique	oral_mother	0.703	7.526	0.81	0.27	1	0.14
X45270_Veillonella__s__dispar	unique	oral_mother	0	6.865	0.332	0	1	0.05
X44165_Streptococcus__s__	unique	oral_mother	0	6.183	0.497	0	1	0.08
X4329957_Prevotella__s__	core	gut&oral_mother&placenta	2.155	4.919	3.183	0.59	0.86	0.49
X4307897_Streptococcus__s__	unique	oral_mother	0.02	4.729	0.189	0.05	0.81	0.03
X4307391_Prevotella__s__melaninogenica	unique	oral_mother	0	4.544	0	0	0.84	0
X3746312_Prevotella__s__nigrescens	unique	oral_mother	0	5.073	0.699	0	0.92	0.11
X269907_Prevotella__s__	unique	oral_mother	0	3.104	0.338	0	0.57	0.05
X2228_Prevotella__s__	unique	oral_mother	0	5.016	0.51	0	0.95	0.08
X1891602_Prevotella__s__nigrescens	unique	oral_mother	0	6.437	0.881	0	0.97	0.14
X1098340_Streptococcus__s__	core	gut&oral_mother&placenta	4.754	13.408	10.006	1	1	0.97
X1097208_Streptococcus__s__	core	gut&oral_mother&placenta	1.007	6.44	3.642	0.45	1	0.51
X1092300_Streptococcus__s__	core	gut&oral_mother&placenta	2.965	7.389	4.422	0.77	1	0.65
X1088134_Streptococcus__s__	core	gut&oral_mother&placenta	1.17	10.613	7.362	0.5	1	0.89
X1085832_Streptococcus__s__	core	gut&oral_mother&placenta	6.974	9.244	4.931	1	1	0.68
X1082539_Streptococcus__s__	pan	gut&oral_mother	2.089	5.846	1.385	0.68	1	0.22
X1068499_Streptococcus__s__	pan	gut&oral_mother	1.233	7.924	1.645	0.45	1	0.22
X1066621_Prevotella__s__melaninogenica	unique	oral_mother	0.569	7.068	1.993	0.18	0.97	0.3
X1061897_Streptococcus__s__	pan	oral_mother&placenta	0.042	6.979	3.719	0.05	1	0.51
X1048420_Streptococcus__s__	unique	oral_mother	0	5.456	1.425	0	1	0.22
X1039405_Streptococcus__s__	unique	oral_mother	0.312	7.582	1.802	0.18	1	0.27
X1027587_Streptococcus__s__	core	gut&oral_mother&placenta	1.61	7.975	3.184	0.55	1	0.43
X1023988_Streptococcus__s__	unique	oral_mother	0	6.212	2.054	0	1	0.32
X1019878_Veillonella__s__dispar	unique	oral_mother	0.147	7.074	1.651	0.05	1	0.24
X1019609_Streptococcus__s__	unique	oral_mother	0.205	6.299	2.343	0.14	1	0.35
X10017_Veillonella__s__parvula	unique	oral_mother	0.147	6.139	1.282	0.05	1	0.24
X1000269_Streptococcus__s__	unique	oral_mother	0.084	6.874	0.642	0.05	1	0.11

Supplementary Figure 9. Differences between vaginal and C-section delivery in microbiome composition

5a) PCoA plots at family and genus level reveal no clustering of the placental microbiome grouped by type of delivery (family: R= 0.019, p = 0.259, genus: R= 0.016, p = 0.272). **5b)** Alpha diversity analyses according to type of delivery (family: shannon $p = 0.929$, chao-1 $p = 0.568$, genus: shannon $p = 0.873$, chao-1, $p = 0.562$) reveal no differences within samples according to type of delivery.

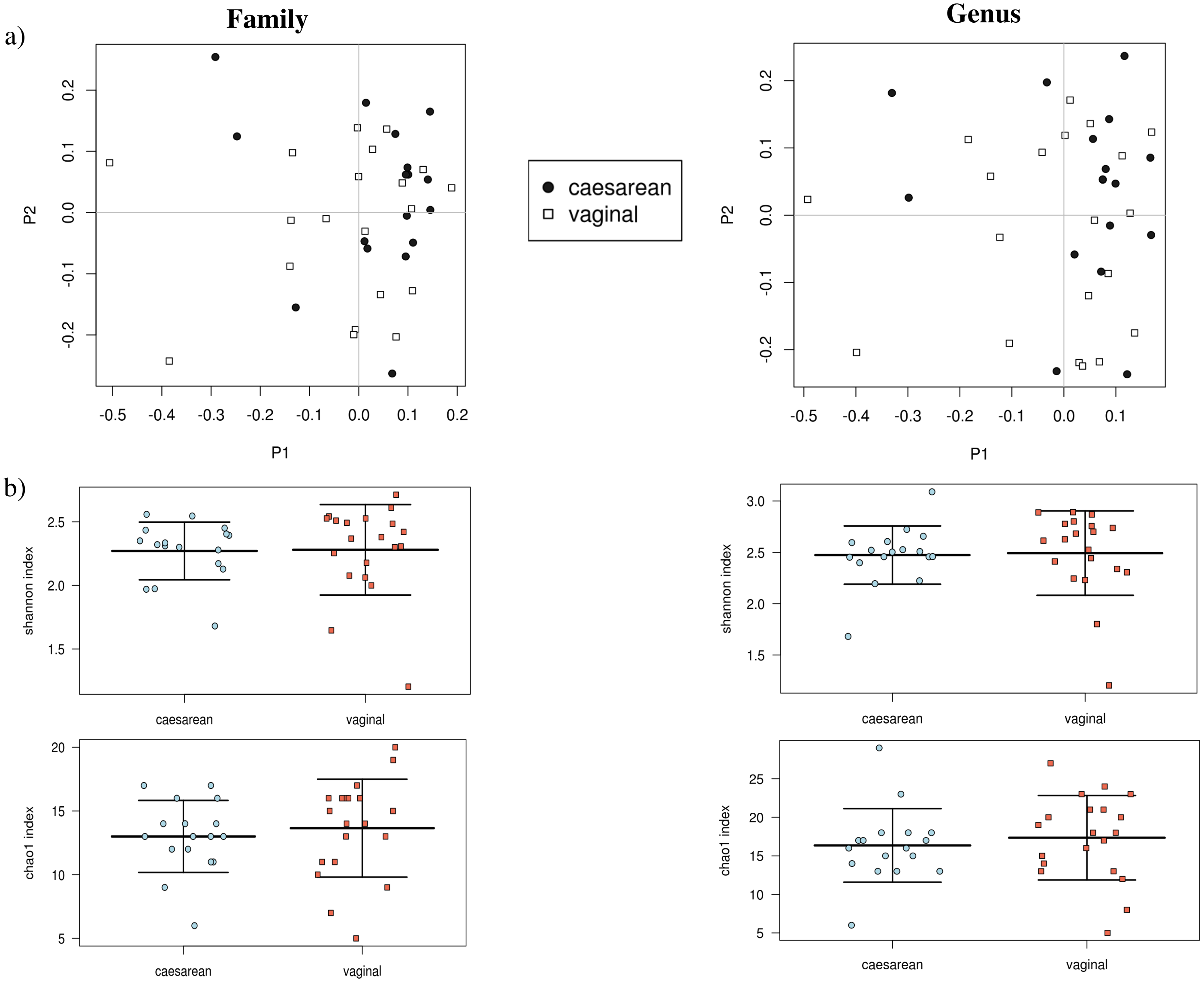

Supplementary Figure 10. Family and genus FDR values assorted by type of delivery. No significant difference in microbial abundance between caesarean and vaginally delivered placenta.

OddsRatio caesarean/vaginal
Top 30 biomarker candidates

	P	FDR	AUC	Log10 Odds ratio [95% CI]
Carnobacteriaceae	0.0056	0.14	0.72	-1.20 [-2.50, -0.40]
Lactobacillaceae	0.0160	0.20	0.73	-0.70 [-1.44, -0.06]
Methylobacteriaceae	0.0590	0.49	0.65	-0.58 [-1.31, 0.05]
Corynebacteriaceae	0.1300	0.73	0.62	-0.49 [-1.22, 0.14]
Oxalobacteraceae	0.1900	0.73	0.63	-0.44 [-1.35, 0.33]
X.Paraprevotellaceae.	0.2000	0.73	0.39	0.21 [-0.38, 0.82]
Actinomycetaceae	0.2100	0.73	0.62	0.61 [-0.09, 1.48]
Neisseriaceae	0.3000	0.73	0.59	-0.15 [-0.73, 0.41]
Staphylococcaceae	0.3100	0.73	0.41	0.21 [-0.38, 0.82]
Bradyrhizobiaceae	0.3600	0.73	0.57	-0.30 [-1.04, 0.36]
Leuconostocaceae	0.3600	0.73	0.44	0.37 [-0.31, 1.13]
Bacilli_NA_NA	0.3600	0.73	0.58	0.05 [-0.51, 0.62]
Prevotellaceae	0.3900	0.73	0.58	0.42 [-0.18, 1.08]
Moraxellaceae	0.4100	0.73	0.57	0.23 [-0.34, 0.81]
Veillonellaceae	0.5300	0.81	0.56	0.07 [-0.66, 0.83]
Pseudomonadaceae	0.5500	0.81	0.56	-0.24 [-0.82, 0.32]
Pasteurellaceae	0.5600	0.81	0.56	0.07 [-0.66, 0.83]
Gemellales_NA	0.5800	0.81	0.45	0.22 [-0.36, 0.81]
Gemellaceae	0.7400	0.90	0.53	-0.14 [-0.81, 0.49]
Gammaproteobacteria_NA_NA	0.7500	0.90	0.52	-0.03 [-0.72, 0.63]
Micrococcaceae	0.7500	0.90	0.53	-0.02 [-0.60, 0.56]
Comamonadaceae	0.9000	0.99	0.49	-0.01 [-0.64, 0.61]
Streptococcaceae	0.9600	0.99	0.49	0.12 [-0.71, 1.05]
Pseudomonadales_NA	0.9800	0.99	0.5	0.04 [-0.53, 0.61]
Streptophyta_f__	0.9900	0.99	0.5	-0.14 [-0.71, 0.43]

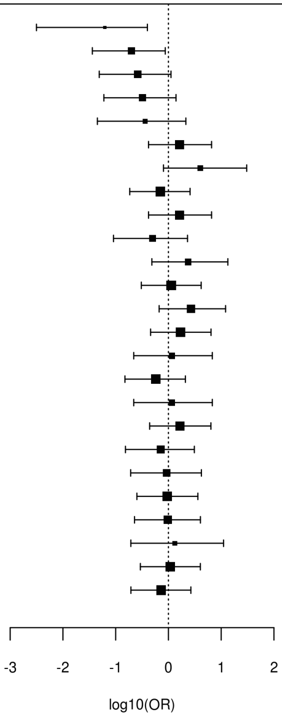

OddsRatio caesarean/vaginal
Top 30 biomarker candidates

	P	FDR	AUC	Log10 Odds ratio [95% CI]
Granulicatella	0.0063	0.31	0.71	-1.20 [-2.50, -0.40]
Lactobacillus	0.0130	0.32	0.74	-0.70 [-1.44, -0.06]
Sphingomonas	0.1100	0.82	0.41	0.77 [-0.12, 2.08]
Corynebacterium	0.1900	0.82	0.6	-0.49 [-1.22, 0.14]
Methylobacterium	0.2000	0.82	0.59	-0.51 [-1.39, 0.21]
Selenomonas	0.2000	0.82	0.43	0.61 [-0.33, 1.94]
Leptotrichia	0.2100	0.82	0.42	0.44 [-0.33, 1.35]
Bacillales_NA_NA	0.2100	0.82	0.57	-0.60 [-1.92, 0.28]
Prevotella	0.2100	0.82	0.62	0.58 [-0.05, 1.31]
Actinomyces	0.3000	0.82	0.6	0.61 [-0.09, 1.48]
Neisseria	0.3000	0.82	0.59	-0.18 [-0.77, 0.40]
Oxalobacteraceae_g__	0.3100	0.82	0.6	-0.44 [-1.35, 0.33]
Bradyrhizobiaceae_NA	0.3200	0.82	0.57	-0.30 [-1.04, 0.36]
Staphylococcus	0.3300	0.82	0.42	0.21 [-0.38, 0.82]
Acinetobacter	0.3300	0.82	0.59	0.23 [-0.34, 0.81]
Ralstonia	0.3500	0.82	0.55	-0.45 [-1.78, 0.49]
Aerococcus	0.3500	0.82	0.55	-0.45 [-1.78, 0.49]
Leuconostoc	0.3600	0.82	0.44	0.37 [-0.31, 1.13]
Streptococcaceae_NA	0.4100	0.88	0.55	-0.27 [-1.18, 0.50]
Microbacterium	0.4900	0.94	0.54	-0.27 [-1.18, 0.50]
Rhizobiales_f___g__	0.5200	0.94	0.47	0.40 [-0.65, 1.76]
Rhodocyclaceae_g__	0.5200	0.94	0.47	0.40 [-0.65, 1.76]
Bacilli_NA_NA_NA	0.5300	0.94	0.56	0.05 [-0.51, 0.62]
Gemellales_NA_NA	0.5500	0.94	0.45	0.22 [-0.36, 0.81]
Pseudomonas	0.5900	0.94	0.55	-0.26 [-0.86, 0.31]
Fusobacterium	0.6300	0.94	0.47	0.29 [-0.55, 1.21]
Bifidobacteriaceae_NA	0.6800	0.94	0.52	-0.25 [-1.61, 0.81]
Achromobacter	0.6800	0.94	0.52	-0.25 [-1.61, 0.81]
Lachnospiraceae_NA	0.6800	0.94	0.52	-0.25 [-1.61, 0.81]
Lactococcus	0.6900	0.94	0.47	0.08 [-0.70, 0.87]

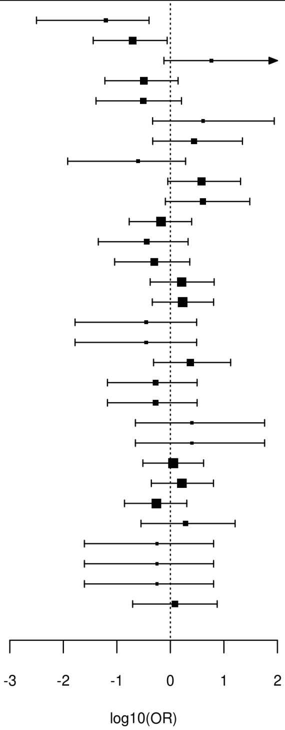

Supplementary Figure 11. Predictive metabolic pathways determined by PiCrust for all three body sites. Metabolic function was compared by LEfSe. Metabolism of tryptophan, fatty acid and benzoate degradation were pathways significantly enriched in the placental microbial communities.

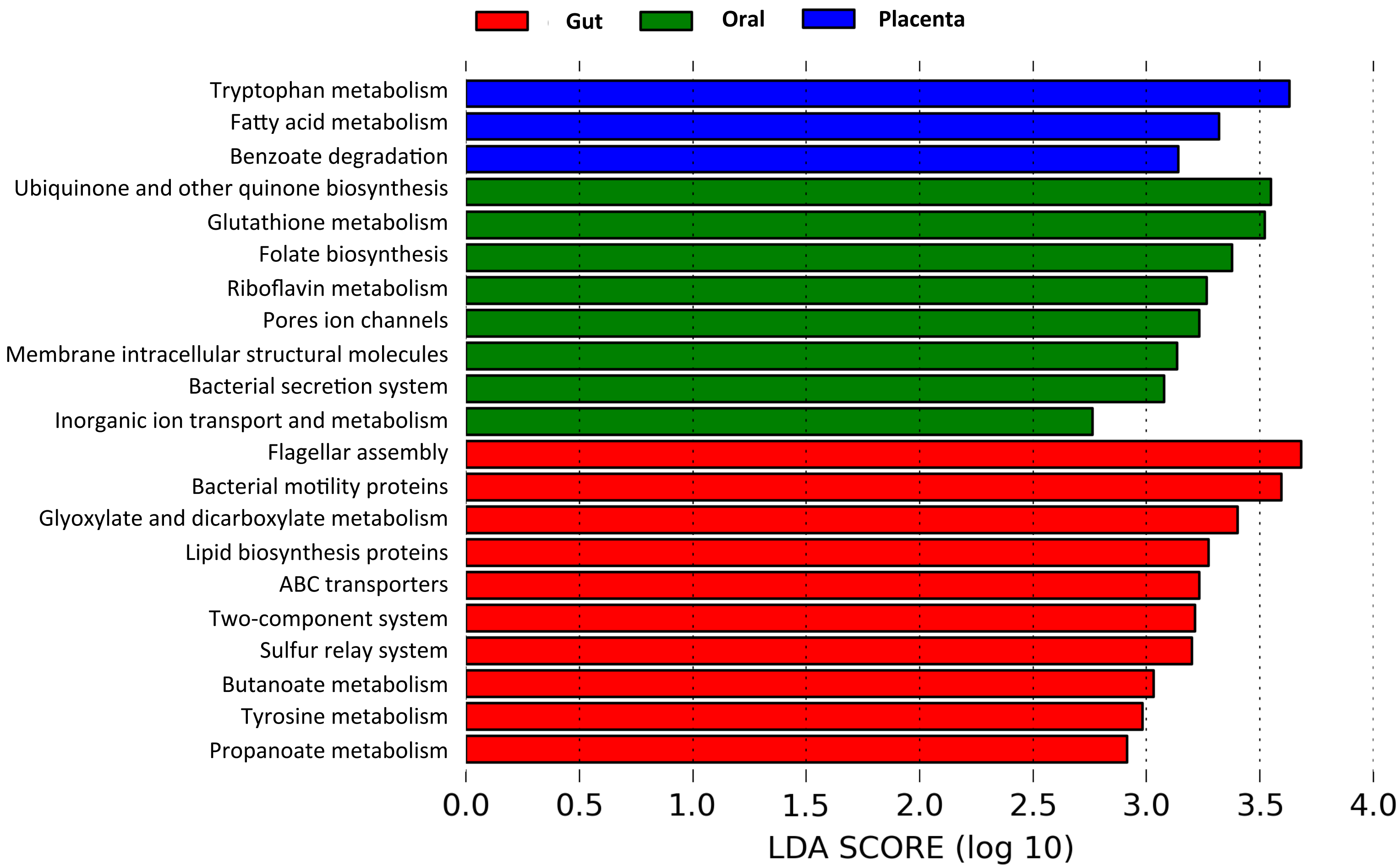

Supplement: Supplementary file 1 — Supplementary information [file 41598_2017_3066_MOESM1_ESM.pdf]
